# The Effect of the Interaction between Abnormal Body Mass Index and Hypertension on the Risk of Type 2 Diabetes

**DOI:** 10.1155/2023/6009414

**Published:** 2023-01-16

**Authors:** Conghui Hu, Yinxia Su, Xiaoyuan Hu, Kun Luo, Alimire Abudireyimu, Yuanyuan Li, Hua Yao

**Affiliations:** ^1^School of Public Health, Xinjiang Medical University, Urumqi, Xinjiang, China; ^2^School of Medical Engineering and Technology, Xinjiang Medical University, Urumqi, Xinjiang, China; ^3^The First Affiliated Hospital of Xinjiang Medical University, Urumqi, Xinjiang, China; ^4^Health Management Institute of Xinjiang Medical University, Urumqi, Xinjiang, China

## Abstract

**Objective:**

Many patients with type 2 diabetes have an abnormal body mass index (BMI) and hypertension together, but few studies on the interaction of the two on the risk of T2DM are reported. We aim to explore the effect of the interaction between abnormal BMI and hypertension on the risk of type 2 diabetes mellitus (T2DM) in Uyghur residents.

**Methods and Results:**

Based on the physical examination data of 27,4819 Uygur residents in Moyu County, a logistic regression model was used to analyze the correlation between BMI abnormality, hypertension, and T2DM disease, and then, the effect of their interaction on the risk of T2DM was evaluated by an additive model and a multiplicative model. The results showed that the detectable rate of T2DM was 5.58%, the proportion of abnormal BMI was 59.49%, and the proportion of hypertension was 25.14%. The risk of T2DM in people with an abnormal BMI and hypertension was higher than that in people with a normal weight and without hypertension, and the difference was statistically significant (*P* < 0.05). The additive model showed that after adjusting for confounding factors such as gender, age, family history of diabetes, abdominal obesity, and alcohol consumption, abnormal BMI and hypertension had a synergistic effect on the risk of T2DM and the evaluation indicators RERI, AP, and S were 0.90 (0.32∼1.49), 0.20 (0.11∼0.30), and 1.36 (1.17∼1.57), respectively. But there was no multiplicative interaction between the two (OR = 0.97, (95% CI: 0.89∼1.06). 3).

**Conclusion:**

The interaction between abnormal BMI and hypertension can increase the risk of T2DM, and improving BMI and controlling blood pressure within the normal range can effectively reduce the risk of T2DM.

## 1. Introduction

Diabetes is a chronic metabolic disease that has become a major global health problem, affecting approximately 422 million adults worldwide [1, 2]. Type 2 diabetes (T2DM) accounts for 90% of all diabetes cases. At present, China is the largest country with diabetes, with a prevalence rate of 11.2% [3]. T2DM is a multifactorial disease caused by genetic and environmental factors. Unhealthy lifestyles, such as a lack of exercise and an unbalanced diet, will increase the incidence of T2DM [4]. There are many influencing factors of T2DM, among which abnormal body mass index (BMI) and hypertension are considered to be the most two important influencing factors of T2DM [5]. It has been reported that BMI, as a measure of human health, has the greatest impact on T2DM [6]. In addition, a large number of studies have shown that hypertension and T2DM usually occur together, and the risk of T2DM in patients with hypertension is higher than that in people without hypertension [7]. In the study of Meng et al., it was shown that the risk of diabetes in hypertensive patients was 1.81 times higher than that of normal people [8]. However, the impact of a single factor on the outcome does not reflect the real impact of the factor because there will be interactions between factors, reducing or increasing the risk of disease. Therefore, the interaction between factors can more realistically reflect the relationship between factors and disease [9]. Current studies have shown that interactions of some other important factors, such as hypertension and dyslipidemia, have a synergistic effect on the occurrence of diabetes [10], but there are few reports on the interaction between abnormal BMI and hypertension on the risk of T2DM [11]. Therefore, this study took Uyghur residents in Moyu County as the research object to explore the effect of the interaction between abnormal BMI and hypertension on the risk of T2DM and to provide a reference for the scientific prevention and control of T2DM.

## 2. Subjects and Methods

### 2.1. Subjects

Based on a cross-sectional design, this study collected data from 274,819 participants in physical examinations from January 2020 to December 2020 in Moyu County, Xinjiang, to determine the prevalence and main risk factors of diabetes among Uyghurs in Moyu County. The inclusion criteria are as follows: 18 years of age and older; Uyghur ethnicity; and complete physical examination data such as body mass index, hypertension, and diabetes. The exclusion criteria are as follows: people under the age of 18, with a body mass index (BMI) < 18.5 kg/m^2^, pregnant women with gestational diabetes mellitus, those using drugs that may alter blood sugar and blood pressure levels, and those with major infectious diseases. All subjects participated voluntarily and signed informed consent.

### 2.2. Methods

#### 2.2.1. Questionnaire

A uniformly designed questionnaire was used to conduct the survey by trained investigators. The content of the questionnaire mainly included general demographic characteristics (such as gender, age, education level, and occupation) and risk factors (such as personal disease history, family history, smoking, drinking, and physical activity).

#### 2.2.2. Physical Examination and Laboratory Tests

Physical examinations were performed on the subjects by unified training staff, including measurements of height, weight, waist circumference, and blood pressure. Each participant had their blood pressure measured twice, at least five minutes, after sitting for a few minutes using a digital blood pressure monitor. The average of these two readings was used in the analysis. Height and weight were measured using standardized methods and calibrated instruments. When participants were barefoot and wearing lightweight clothing, their weight was measured using a calibrated health meter digital scale. Laboratory tests mainly include the measurement of total cholesterol (TC), triglyceride (TG), low-density lipoprotein cholesterol (LDL-C), and high-density lipoprotein cholesterol (HDL-C).

#### 2.2.3. Diagnostic Criteria

(1) The diagnostic criteria for diabetes were based on the diagnostic criteria of the “China Guidelines for the Prevention and Treatment of Type 2 Diabetes (2020 Edition)” [3]: fasting blood glucose (FPG) ≥ 7.0 mmol/L (126 mg/dL), or has been diagnosed with diabetes, or is taking hypoglycemic drugs or insulin injection; (2) diagnostic criteria for hypertension [12]: systolic blood pressure ≥ 140 mmHg and/or diastolic blood pressure ≥ 90 mmHg, or those who have been diagnosed with hypertension or who are taking antihypertensive drugs; (3) abnormal BMI [13]: BMI ≥ 24 kg/m^2^; (4) dyslipidemia [14]: cholesterol (TC) ≥ 6.22 mmol/L (111.96 mg/dL) or triglyceride (TG) ≥ 2.25 mmol/L (40.5 mg/dL) or low-density lipoprotein cholesterol (LDL-C) ≥ 4.14 mmol/L (74.52 mg/dL) or high-density lipid protein cholesterol (HDL-C) < 1.0 mmol/L (18 mg/dL); (5) abdominal obesity [13]: women: waist circumference ≥ 85 cm; men: waist circumference ≥ 90 cm; (6) family history of diabetes: it means that at least one of the first-degree relatives (parents, siblings, or children) of the research subjects has diabetes.

### 2.3. Statistical Analysis

The data were analyzed with SPSS 23.0 software. Normally distributed measurement data were expressed as mean ± standard deviation, nonnormal distribution was expressed as median (interquartile range), comparison between groups was expressed by a *t*-test or rank sum test, categorical data were expressed by quantity (rate), and the chi-square test was used for comparison between groups; *P* < 0.05 indicated a statistically significant difference. A logistic regression model assessed the influence of abnormal body mass index and hypertension on the prevalence of T2DM. The additive interaction was analyzed using the Excel table prepared by Andersson [15]. Evaluation indicators include the relative excess risk ratio (RERI), attributable ratio (AP), and interaction index (S).

The indicator calculation formula is as follows:

(1) $$\begin{array}{c} RERI={RR}_{11}-{RR}_{10}-{RR}_{01}+1\text{,}\\ AP=\frac{RERI}{{RR}_{11}}\text{,}\\ S=\frac{\left({RR}_{11}-1\right)}{\left[\left({RR}_{01}-1\right)+\left({RR}_{10}-1\right)\right]}\text{.} \end{array}$$

Among them, RR_11_ represents the OR value when two factors exist at the same time, and RR_10_ and RR_01_ represent the OR value when only one of the two factors exists. If there is no additive interaction between the two factors, the confidence intervals for RERI and AP should contain 0, and those for S should contain 1.

The metrics used to evaluate the multiplicative interaction are *OR*=RR_11_/(RR_01_ × RR_10_). If there is no multiplicative interaction between the two factors, the confidence interval for the OR value should contain 1.

## 3. Results

### 3.1. The Basic Characteristics of the Research Object

The detectable rate of T2DM in 274 819 subjects was 5.58% (15 338/274 819), of which the detectable rate of T2DM in men was 5.95% (7 563/127 043) and in women was 5.26% (7 775/147 776). There were significant differences between the case group and the control group in gender, age, education level, family history of diabetes, abdominal obesity, abnormal body mass index, dyslipidemia, hypertension, and drinking (*P* < 0.05) (see Table 1).

### 3.2. Logistic Regression Analysis of Abnormal BMI and Hypertension

A logistic regression analysis was conducted with T2DM as the dependent variable and abnormal BMI and hypertension as independent variables. The results showed that there were significant differences in abnormal BMI and hypertension (*P* < 0.01). The results of multivariate logistic regression analysis showed that there were still significant differences in patients with abnormal BMI (OR = 1.551) and patients with hypertension (*P* < 0.01), as shown in Table 2.

### 3.3. Interaction Analysis of Abnormal BMI and Hypertension on T2DM

#### 3.3.1. Additive Interaction of Abnormal BMI and Hypertension on T2DM

With normal BMI and no hypertension as the reference group, the OR value of hypertension alone was 3.03 after adjusting for influencing factors. OR value was 1.51 when BMI abnormality existed alone after adjusting for influencing factors. The OR value was 4.44 for the combination of abnormal BMI and hypertension. The adjusted OR values are all lower than the unadjusted OR values, as shown in Table 3.

Abnormal BMI and hypertension have a synergistic effect on the risk of T2DM. After adjusting for influencing factors, the morbidity effect of BMI abnormality and hypertension was 1.36 times greater than that of BMI abnormality and hypertension alone (S). The prevalence rate caused by the combination of the two factors was 0.90 times that caused by other factors (RERI). Among all T2DM cases, 0.20% were attributed to the synergistic effect of abnormal BMI and hypertension (AP (%)), as shown in Table 4.

#### 3.3.2. Multiplicative Interaction of Abnormal BMI and Hypertension on T2DM

Abnormal BMI, hypertension, and the product of the two were introduced into the logistic regression model for multiplicative interaction analysis. Model 1 results showed that abnormal BMI and hypertension had a negative multiplicative interaction with T2DM (OR = 0.81, 95% CI: 0.75∼0.88). The results of Model 2 showed no multiplicative interaction between abnormal BMI and hypertension on T2DM (OR = 0.97, 95% CI: 0.89∼1.06), as shown in Table 5.

## 4. Discussion

This study found that the detectable rate of T2DM in the Uyghur health check-up population in Moyu County was 5.95%, which was lower than the current national diabetes prevalence rate [3]. The detectable rates of hypertension and abnormal body mass index were 25.14% and 59.49%, respectively. The prevalence of hypertension, dyslipidemia, and abnormal body mass index in the T2DM group was 62.65%, 44.22%, and 79.47%, respectively. The current findings support the high detectable rates of diabetes, hypertension, overweight, and obesity among the Uyghur population in Moyu County and highlight the urgent need for early detection and prevention measures. In addition, the interaction analysis results of abnormal BMI and hypertension showed that there was a synergistic effect between the two before and after adjusting for gender, age, education level, family history of diabetes, abdominal obesity, dyslipidemia, and alcohol consumption; that is, when abnormal BMI and hypertension exist at the same time, the risk of T2DM is higher than the sum of abnormal BMI and hypertension alone. However, there was no multiplicative interaction between adjusted BMI abnormality and hypertension on T2DM.

Some studies have shown that an abnormal BMI and high blood pressure are risk factors for diabetes [6], which is consistent with the findings of this study. The accumulation of body fat in people with an abnormal BMI causes hyperinsulinemia and insulin resistance, which reduce the utilization of glucose by muscle and other tissues, resulting in decreased glucose tolerance and the occurrence of T2DM [5]. Moreover, the risk of diabetes in obese people is 1.998 times that of nonobese people [16]. Studies have shown that obesity accounts for more than 80% of the physiological pathology of metabolic syndrome, so losing weight is an important step to reducing the incidence of T2DM, especially for young people [17]. Therefore, the more weight loss an obese or overweight person has, the greater the chance of remission of diabetes [18]. In addition, studies have shown that the risk of developing diabetes in hypertensive patients is 2.5 times that of nonhypertensive people [19]. The association between hypertension and T2DM can be explained by hyperglycemia, insulin resistance, and dyslipidemia, all of which contribute to the atherosclerotic process. This process can lead to vascular stenosis and increased peripheral arterial resistance, hallmarks of hypertension [20]. Studies have shown that T2DM is more causally associated with higher systolic blood pressure than diastolic blood pressure, and significantly reducing systolic blood pressure in T2DM patients can reduce the risk of cardiovascular mortality and other risks [21]. In this study, after adjusting for confounders, the risk of T2DM in the abnormal BMI group was 1.55 times higher than that in the normal BMI group. Compared with subjects with normal blood pressure, subjects with hypertension had a 3.00-fold increased risk of T2DM. It shows that effective control of blood pressure and improvement of BMI are of great significance for preventing the occurrence and development of diabetes.

Studies have reported that a family history of diabetes and dyslipidemia [22], hypertension and dyslipidemia [9], and a family history of diabetes and the waist-height ratio [23] have synergistic effects on the occurrence of diabetes. However, there are few reports on the interaction between abnormal BMI and hypertension and the risk of T2DM. In a study on the effect of the interaction of drinking and smoking on the incidence of diabetes, it was shown that smoking cessation and drinking abstinence did not have additive and multiplicative interactions on diabetes; however, smoking increased the risk of diabetes, and alcohol consumption was found to increase the risk of diabetes [24]. In another study, family history and obesity were shown to have a synergistic interaction on the pathogenesis of diabetes, and the independent effects of the two were not negligible [25]. In normotensive populations, when a family history of diabetes and hyperlipidemia coexist, there may also be a synergistic effect on diabetes [26]. The abovementioned studies have shown that diseases such as hypertension, family history, and dyslipidemia play a greater or lesser role in the risk of diabetes, and the comprehensive incidence of diseases has a greater impact on diabetes than the harm of one complicating disease. The advantages of this study include the large sample size and the fact that the T2DM cases in this study were diagnosed by clinicians. However, this study also has some limitations; that is, it is a cross-sectional survey and cannot determine the causal relationship between influencing factors and disease, which needs to be verified in future cohort studies.

## 5. Conclusion

In conclusion, abnormal BMI and hypertension have a synergistic effect on the risk of T2DM. Both BMI and blood pressure are controllable factors, so it is recommended that residents strengthen the management of blood pressure and BMI through such measures as reasonable exercise, a healthy diet, smoking cessation, and alcohol cessation, so as to prevent the occurrence and development of T2DM.

## Figures and Tables

**Table 1:** Analysis of the basic characteristics of the research object.

Indexes	Case group (*n* (%))	Control group (*n* (%))	*x* ^2^	*P*
Age (year)			8496.76	<0.01
<40	2 845 (18.55)	147 094 (56.69)		
≥40	12 493 (81.45)	112 387 (43.31)		

Gender			62.03	<0.01
Male	7 563 (49.31)	119 480 (46.05)		
Female	7 775 (50.69)	140 001 (53.95)		

Educational level			50.91	<0.01
Below high school	14 144 (92.22)	234 746 (90.47)		
High school and above	1 194 (7.78)	24 735 (9.53)		

Drinking			7.16	0.01
No	14 802 (5.60)	249 297 (94.40)		
Yes	531 (5.00)	10 096 (95.00)		

Family history of diabetes			279.66	<0.01
No	14 788 (96.41)	255 007 (98.28)		
Yes	550 (3.59)	4 474 (1.72)		

Abdominal obesity			3510.39	<0.01
No	3 519 (22.94)	123 216 (47.49)		
Yes	11 819 (77.06)	136 265 (52.51)		

Abnormal BMI			2690.40	<0.01
No	3 149 (20.53)	108 177 (41.69)		
Yes	12 189 (79.47)	151 304 (58.31)		

Hypertension			12 147.01	<0.01
No	5 728 (37.35)	199 999 (77.08)		
Yes	9 610 (62.65)	59 482 (22.92)		

Dyslipidemia			3475.55	<0.01
No	8 556 (55.78)	199 313 (76.78)		
Yes	6 782 (44.22)	60 168 (23.22)		

Note. BMI: body mass index.

**Table 2:** Logistic analysis of abnormal BMI, hypertension, and T2DM.

	Single factor^a^		Multivariate^b^	
	OR (95% CI)	*P*	OR (95% CI)	*P*
Abnormal BMI				
No	1.00		1.00	
Yes	2.77 (2.66∼2.88)	<0.01	1.55 (1.48∼1.63)	<0.01

Hypertension				
No	1.00		1.00	
Yes	5.64 (5.45∼5.84)	<0.01	3.00 (2.89∼3.12)	<0.01

Note. a: not adjusted; b: adjusted for gender, age, educational level, abdominal obesity, family history of diabetes, dyslipidemia, and alcohol consumption. BMI, body mass index; OR, odds ratio; CI, confidence interval.

**Table 3:** Additive interaction of abnormal BMI and hypertension on T2DM.

Abnormal BMI	Hypertension	Control group (*n*)	Case group (*n*)	Model 1	Model 2
				OR (95% CI)	OR (95% CI)
No	No	91 495	1 516	1.00	1.00
No	Yes	16 682	1 633	5.91 (5.50∼6.35)	3.03 (2.81∼3.27)
Yes	No	108 504	4 212	2.34 (2.21∼2.49)	1.51 (1.41∼1.61)
Yes	Yes	42 800	7 977	11.25 (10.64∼11.90)	4.44 (4.16∼4.75)

Note. Mode 1: not adjusted; Mode 2: adjusted for gender, age, educational level, abdominal obesity, family history of diabetes, dyslipidemia, and alcohol consumption. BMI, body mass index; OR, odds ratio; CI, confidence interval.

**Table 4:** Evaluation metrics for additive interactions.

Models	RERI	AP (%)	SI
Model 1	4.00 (2.47∼5.53)	0.36 (0.28∼0.43)	1.64 (1.45∼1.86)
Model 2	0.90 (0.32∼1.49)	0.20 (0.11∼0.30)	1.36 (1.17∼1.57)

Note. RERI, relative excess risk due to interaction; AP, attribution proportion; SI, index of interaction.

**Table 5:** Multiplicative interaction of abnormal BMI and hypertension on T2DM.

Variables	Model 1		Model 2	
	OR value	95% CI	OR value	95% CI
Abnormal BMI	2.34	2.21∼2.49	1.51	1.41∼1.61
Hypertension	5.91	5.50∼6.35	4.44	4.16∼4.75
Abnormal BMI^*∗*^hypertension	0.81	0.75∼0.88	0.97	0.89∼1.06

Note. Mode 1: not adjusted; Mode 2: adjusted for gender, age, educational level, abdominal obesity, family history of diabetes, dyslipidemia, and alcohol consumption. BMI, body mass index; OR, odds ratio; CI, confidence interval.

## Data Availability

The datasets used to support the findings of this study are available from the corresponding author upon reasonable request.
